# Role of Regulatory T Cells in Tumor-Bearing Mice Treated with Allo-Hematopoietic Stem Cell Transplantation Plus Thymus Transplantation

**DOI:** 10.1155/2018/7271097

**Published:** 2018-07-02

**Authors:** Naoki Hosaka

**Affiliations:** ^1^Department of Pathology, Fuchu Hospital, 1-10-7, Hiko-cho, Izumi, Osaka 594-0076, Japan; ^2^Department of Public Health, Kansai Medical University, 2-5-1 Shin-machi, Hirakata, Osaka 573-1010, Japan

## Abstract

We recently developed a new allogeneic hematopoietic stem cell transplantation method (allo-HSCT) combined with thymus transplantation (TT) from the same donor (allo-HSCT + TT). This method induces elevated T cell function with mild graft-versus-host disease (GVHD) in comparison to conventional HSCT alone and HSCT + donor lymphocyte infusion (DLI). This new method is effective against several intractable diseases, including malignant tumors, for which conventional treatments are ineffective. Regulatory T (T_reg_) cells play an important role in the enhanced graft-versus-tumor (GVT) effect and reduction of GVHD, thus leading to longer survival. Replacement and reduction of elevated T_reg_ cells by donor-derived allo-T_reg_ cells from the transplanted thymus may play one of crucial roles in the effect. This review discusses the role of T_reg_ cells in a tumor-bearing mouse model treated with allo-HSCT + TT.

## 1. Introduction

We recently developed a new allogeneic hematopoietic stem cell transplantation method (allo-HSCT) in conjunction with thymus transplantation (TT) from the same donor (allo-HSCT + TT) [1–11]. This method results in elevated T cell function with mild graft-versus-host disease (GVHD) compared to HSCT alone or HSCT + donor lymphocyte infusion (HSCT + DLI) [8]. The mechanism underlying these effects involves CD4^+^ FoxP3^+^ regulatory T (T_reg_) cells, which suppress immune activity and prevent autoimmunity and GVHD [12, 13]. The percentages of these cells in CD4^+^ T cells are intermediate between HSCT alone and HSCT + DLI, while the opposite is true for the percentage of CD4^+^ FoxP3^−^ effector T (T_eff_) cells. There are two main ways of producing T_reg_ cells—that is, from the thymus (as naturally occurring T_reg_, nT_reg_) and from peripheral cells (inducible T_reg_, iT_reg_) [14, 15]. We observed that not only the number of T cells but also the quantity of T cell receptor rearrangement excision circles (TREC) [8], which reflect production of T cells from the thymus, are increased in HSCT + TT. Although we did not purify the T_eff_ and T_reg_ cells in TREC analysis, we suggest that both naive cells are produced from the transplanted thymus and move to the periphery because of fundamentally similar mechanisms of them for those cells [16].

This method showed efficacy against several intractable diseases and conditions, such as autoimmune diseases in aging and radioresistant hosts [2, 3], exposure to supralethal irradiation [4], multiple-organ transplantation from different donors [5], type 2 diabetes mellitus [6], low hematopoietic stem cell (HSC) number or low dose of irradiation [7], and malignant tumors, including leukemia [8–11]. Malignant tumor-bearing mice treated with allo-HSCT + TT showed a strong graft-versus-tumor (GVT) effect but weak GVHD compared with HSCT alone and HSCT + DLI. These effects may involve replacement and reduction of the elevated T_reg_ cells by allo-T_reg_ cells.

The regulation of T_reg_ cells was suggested to be one mechanism of action of immunotherapy for cancer, and this has been examined in clinical trials [17]. It may also be applicable under allo-HSCT + TT. We review and discuss the utility of T_reg_ cells for treatment of cancer.

## 2. Main Text

### 2.1. Review

#### 2.1.1. Theory of HSCT + TT with T_reg_ Cells

**Figure 1:**
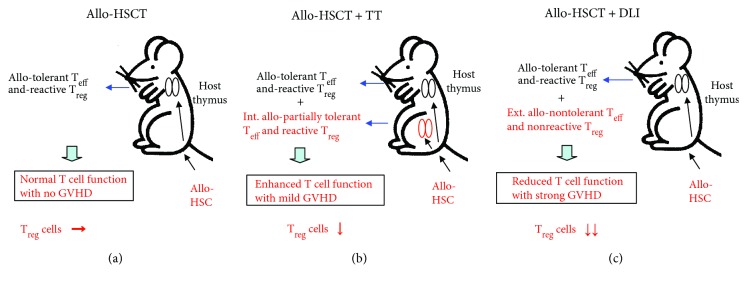
Theory of allo-HSCT + TT. In the case of conventional allo-HSCT (a), allo-T_eff_ and T_reg_ cells develop, are tolerated, and react in the host thymus. No GVHD occurs. The proportion of T_reg_ cells is comparable to that in normal mice. In the case of allo-HSCT + DLI (c), allo-nontolerant T_eff_ and nonreactive T_reg_ cells are externally supplied, and strong GVHD is induced with reduction of T cell function. The proportion of T_reg_ cells is markedly decreased. In the case of allo-HSCT + TT, the allo-T_eff_ and T_reg_ cells develop internally in the allo-transplanted thymus. The T cells show partial tolerance and reaction with the host, and only mild GVHD occurs with elevation of T cell function (b). The proportion of T_reg_ cells decreases slightly. Figure 1 is modified from Hosaka [1], under the Creative Commons Attribution License/public domain.

First, we present the theory of allo-HSCT + TT [1, 8]. This method makes use of intra-bone marrow-bone marrow transplantation (IBM-BMT) for HSCT, which involves the direct injection of HSC into the bone marrow cavity, and results in superior engraftment of donor cells and reduced incidence of GVHD with mesenchymal stem cells (MSC) [18–20]. In the case of conventional allo-HSCT, allo-HSC are transplanted into the host, and allo-T cells develop in the host thymus (Figure 1(a)). The T_eff_ cells induce tolerance toward the host with thymic antigen-presenting cells (APC) and/or epithelial cells (TEC) [21]. Host-reactive T_reg_ cells are also reacted with host thymic dendritic cells (DC) [22]. Neither T cell type induces apparent GVHD, and the proportion of T_reg_ cells is comparable to that in normal mice. In contrast, nontolerant allo-T_eff_ and nonreactive T_reg_ cells are externally supplied in the case of HSCT + DLI, resulting in strong GVHD (Figure 1(c)). As this results in expansion of T_eff_ cells and little proliferation of T_reg_ cells, the proportion of T_reg_ cells is markedly reduced. In HSCT + TT (Figure 1(b)), allo-T_eff_ and T_reg_ cells develop internally from the transplanted allo-thymus in the host. The T_eff_ and T_reg_ cells are partially tolerant and reactive to the host, which was suggested to show a low response in mixed lymphocyte reaction, resulting in low GVHD [8]. Under these conditions, most allo-T_eff_ cells derived from the transplanted thymus are in the naïve state and may not expand well to host antigens. The T_reg_ cells also suppress activation of naïve cells by deprivation of activation signals [23]. Therefore, T_reg_ cells may play a role in allo-HSCT + TT. Nonetheless, the degree of inhibition may be insufficient, leading to mild GVHD with a slight decrease in the proportion of T_reg_ cells.

#### 2.1.2. Effects of Allo-HSCT + TT in Tumor-Bearing Mice and the Dynamics of T_reg_ Cells

Next, we describe the effects of HSCT + TT in tumor-bearing mice and the dynamics of T_reg_ cells (Table 1). Non-tumor-bearing mice without treatment, or those treated with HSCT alone, with HSCT + adult thymus (AT) transplantation, or with HSCT + DLI, were used as representative non-tumor-bearing controls (Figure 1, group 1). The tumor-bearing mice showed an increase in T_reg_ cell number with inducible T_reg_ cells [24]. Treatment with HSCT in the early phase of tumor progression (group 2a) resulted in a reduction in the proportion of T_reg_ cells among CD4^+^ T cells, although they were still elevated compared with non-tumor-bearing mice. In HSCT + AT treatment, the proportion of T_reg_ cells decreased further and was comparable to the level in non-tumor-bearing mice. The mice showed the longest survival with strong GVT effects and mild GVH effects.

The functions of the transplanted thymus from AT, newborn thymus (NT), and fetal thymus (FT) in mice treated with HSCT were compared (group 2b, c), as the functions differ between ages. The proportion of T_reg_ cells did not change with any type of HSCT alone or HSCT + TT, and all of the HSCT + TT mice showed strong GVT and longer survival compared to nontreated controls or those treated with HSCT alone. However, the GVT effects in HSCT + NT or FT transplantation were greater than those of HSCT + AT transplantation, and the survival was longest in HSCT + NT transplantation. These animals showed the highest levels of IFN*γ* and effector memory (EM) T cells and the lowest numbers of myeloid suppressor cells [10].

In advanced tumors (group 3), nontreated tumor-bearing mice showed marked elevation of T_reg_ cell number. HSCT + TT reduced the T_reg_ cell number to a greater extent than did HSCT alone and inhibited lung metastasis leading to the longest survival, although the T_reg_ cell level did not decrease to normal and there was no significant regression of the primary tumor [9].

The results with regard to T_reg_ cells in leukemia-bearing mice in group 4 were similar to those of non-tumor-bearing controls (group 1). The T_reg_ cell number in the HSCT + AT transplantation group was intermediate between those of HSCT alone and HSCT + DLI, and HSCT + AT transplantation yielded the longest survival with the greatest graft-versus-leukemia (GVL) effect and attenuated GVHD [11].

### 2.2. Discussion

**Figure 2:**
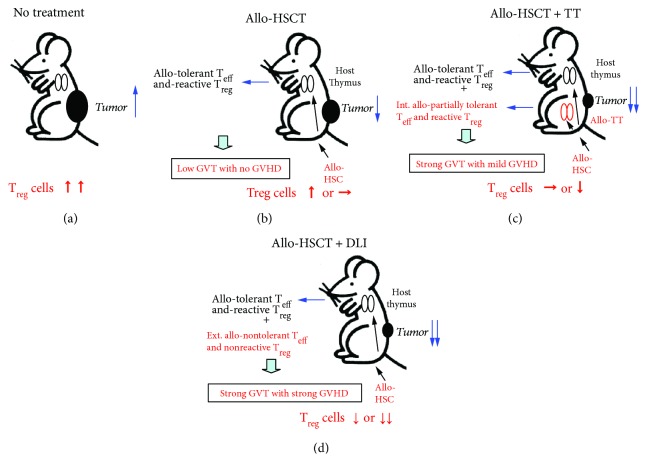
Theory of allo-HSCT + TT for tumors. Untreated controls bearing tumor tissue showed increased numbers of T_reg_ cells, including iT_reg_ cells, with tumor progression (a). In the case of conventional allo-HSCT (b), the allo-T_eff_ and T_reg_ cells develop with tolerance and reactivity to the host in the thymus. A low GVT effect is then induced with no/minimal GVHD. Mild tumor regression is induced compared with untreated controls (a). The proportion of T_reg_ cells still increases slightly or is at the normal level. In the case of allo-HSCT + DLI (d), nontolerant and nonreactive allo-T_eff_ and T_reg_ cells are supplied externally, and a strong GVT effect occurs with strong GVHD. The proportion of T_reg_ cells decreases either slightly or markedly. In the case of allo-HSCT + TT (c), internally allo-partially tolerant T_eff_ and reactive T_reg_ are induced from the transplanted thymus. As a result, strong GVT occurs with mild GVHD. The proportion of T_reg_ cells decreases slightly or is at the normal level.

HSCT + TT is a valuable method for treatment of cancer, and T_reg_ cells play a crucial role in mediating the effects of this method. As shown in Figure 2, T_reg_ cell number was elevated in untreated hosts bearing tumors and increased with tumor progression (Figure 2(a)). Tumor cells produce TGF*β*, which induces iT_reg_ cells leading to inhibition of immune reaction against cancer [25, 26]. Allo-HSCT alone showed a mild GVT effect by allo-reaction with a slight reduction in T_reg_ cell number compared to syngeneic HSCT [9] (Figure 2(b)). Additional transplantation of thymus grafts showed a further GVT effect with further reduction in T_reg_ cell number (Figure 2(c)). The level of GVT was comparable to that from HSCT + DLI leading to long survival, although animals treated with HSCT + DLI showed higher GVHD and shorter survival with lower T_reg_ cell number (Figure 2(d)). These findings were consistent with an important role of T_reg_ cells in inducing strong GVT effects and mild GVH effects in HSCT + TT [27].

Thymic function is known to differ according to age [28–30]. Therefore, we next performed comparisons between fetal, newborn, and adult thymic grafts. Although the proportion of T_reg_ cells was the same in all of these groups, NT showed the best effect with regard to GVT and survival. This may have been related to its strong reduction of myeloid suppressor cells, which inhibit immune activity [31, 32], and elevated production of effector memory T cells and IFN*γ* [10]. Although the detailed mechanism is not yet clear, it is possible that NT shows the highest function of T cell production among the thymus grafts [4].

Mice bearing advanced tumors showed further elevation of T_reg_ cell level. Therefore, the level was not normalized by HSCT + TT, and the primary tumor did not show significant regression. Nonetheless, they showed inhibition of metastasis and long-term survival, suggesting that this method is still effective with regard to GVT on newly developed tumor cells.

Although mice bearing leukemia showed similar results, those treated with either HSCT + TT or DLI showed long survival with complete remission of tumor cells by donor-derived cells. Therefore, the latter may have reduced production of T_reg_ cells from the tumor and/or thymus graft leading to greater GVHD than the former.

Some of our data were based on conversion of T_eff_ cells to T_reg_ cells in the tumor microenvironment [24, 25]. However, a recent study involving analysis of TCR repertoires in a mouse model using chemical carcinogen-induced fibrosarcoma showed that such conversion does not occur [33]. Although the reason for the discrepancy remains unclear, it is possible that the properties of the tumors were different between the studies. Generally, cancers develop with gradual accumulation of gene mutations and express cancer antigens accompanied by immune reactions involving T_eff_/T_reg_ interaction and/or conversion. The specific carcinogen-induced tumor is unknown to be the same condition. Further studies are required using several cancer models developed with different mechanisms.

**Figure 3:**
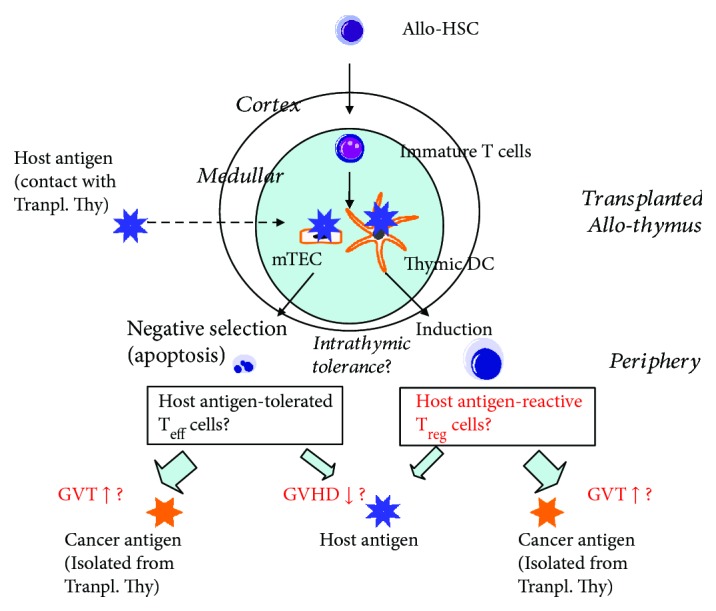
Hypothesis for immune regulation of malignant tumor by allo-HSCT + TT. The host antigen comes into direct contact with the transplanted thymus, whereas cancer antigens are relatively isolated from the transplanted thymus. Therefore, host antigen-specific T_reg_ cells may be induced intrathymically to a greater extent than those specific to cancer antigens with thymic DC. Conversely, T_eff_ cells are tolerant toward host antigens, but not cancer antigens with medullary TEC (mTEC), yielding the same results. This may result in strong GVT with mild GVHD. Figure 3 is modified from Hosaka [1], under the Creative Commons Attribution License/public domain.

The mechanism underlying the production of T_reg_/T_eff_ cells from allo-TT has not been clarified under cancer-bearing conditions. As shown in Figure 3, the host antigen comes into direct contact with the transplanted thymus from renal capsules, in which it is translated, whereas cancer antigens are relatively isolated from and do not come into direct contact with the transplanted thymus, as tumor cells were transplanted subcutaneously into the backs of the experimental animals [8–10]. This may lead to host antigen-specific T_reg_ cells being superior to cancer antigens for inducing thymic DC in the transplanted thymus, likewise tolerance with intrathymical administration [34], which may result in inhibition of GVHD, but not GVT. Conversely, T_eff_ cells are tolerant toward host antigens, but not cancer antigens, yielding the same results.

The regulation of T_reg_ cells is regarded as a suitable target for immune therapy in human cancers and has been the subject of several clinical trials. The main purpose is effective deletion of T_reg_ cells to enhance immune function against tumor cells [35]. Treatment with antibodies for cytotoxic T lymphocyte-associated antigen (CTLA) 4, which is expressed constitutively in T_reg_ cells, or CC chemokine receptor (CCR) 4, which is expressed in activated T_reg_ cells, has been shown to have beneficial effects against melanoma, renal cell carcinoma, and bladder cancer [36–39]. Administration of metronomic cyclophosphamide, which reduces highly proliferative T_reg_ cells, has beneficial effects on advanced cancers and metastatic breast cancers [40, 41]. As a side effect, blockade of immune checkpoints, such as CTLA-4 and programmed death (PD) 1, may induce serous autoimmune diseases [42–44]. In such cases, targeted depletion of tumor-infiltrating T_reg_ cells has been suggested [45]. In addition, it is also important to assess susceptibility to autoimmune diseases in patients with human lymphocyte antigen (HLA) haplotype and monitoring the number of T_reg_ cells.

Although allo-HSCT + TT shows the opposite immune reaction to these reports, the basic theory and points of note are similar. Although allo-T cells can induce GVT, the method using the above antibodies may be applicable in cases when the immune reaction is insufficient. In contrast, if the reaction is too strong with GVHD as autoimmune disease, iT_reg_ can be induced in vivo and/or ex vivo by treatment with IL-10 and/or TGF*β* and subsequently transferred to the host [46, 47].

Taken together, allo-HSCT + TT is effective for treatment of malignant tumors, and T_reg_ cells may play one of crucial roles in the regulation. Among TT from various ages, NT showed the best functionality. Therefore, regenerative thymus tissue would be better than surgically obtained tissue. With recent progress in engineering for thymus regeneration [48–52], HSCT + TT may be useful as next-generation therapy for treatment of human cancer with control of T_reg_ cells.

## 3. Conclusions

T_reg_ cells play a crucial role in allo-HSCT + TT for treatment of malignant tumors. Additional control and regulation of T_reg_ cells may lead to better results, and this method may be applicable to human cancer.

## Figures and Tables

**Table 1:** Effects of T_reg_ cells in tumor-bearing mice treated with allo-HSCT + TT

Group	TT	Comparison of T_reg_ cells^∗^				Effect of HSCT + TT^§^	Ref.
		No treated^#^	HSCT	HSCT + TT	HSCT + DLI		
1. No tumor	AT		→	↓	↓↓	aGVH, elevated T cell function	[1, 8]
2a. Early tumor	AT	↑↑	↑	→	ND	GVT^a^, LS^a^,	[8, 10]
2b. Same as above	NT	↑↑	↑	→	ND	GVT^b^, LS^b^, IFNγ^b^, EM T cells^b^, and MS cells^c^	[10]
2c. Same as above	FT	↑↑	↑	→	ND	GVT^b^, LS^d^	[10]
3. Advanced tumor	FT	↑↑↑	↑↑	↑	ND	Inhibition of metastasis, LS	[9]
4. Leukemia	AT	ND	→	↓	↓↓	GVL, aGVH, and LS	[11]

^∗^% of FoxP3^+^ cells in CD4^+^ T cells compared with non-tumor-bearing mice: no change, →: mild increase, ↑; moderate increase, ↑↑; strong increase, ↑↑↑; slight decrease, ↓; moderate decrease, ↓↓. ^#^Host-derived cells. ^§^Compared with HSCT and/or HSCT + DLI in the same group. ^a^3rd in group 2, ^b^1st in group 2, ^c^lowest in group 2, ^d^2nd in group 2. AT: adult thymus; NT: newborn thymus; FT: fetal thymus; aGVH: attenuated graft-versus-host; GVT: graft-versus-tumor; LS: longest survival; EM: effector memory; MS: myeloid suppressor; GVL: graft-versus-leukemia; ND: not determined.

## Abbreviations

allo-HSCT: Allogeneic hematopoietic stem cell transplantation
TT: Thymus transplantation
AT: Adult thymus
DLI: Donor lymphocyte infusion
FT: Fetal thymus
NT: Newborn thymus
GVHD: Graft-versus-host disease
GVT: Graft versus tumor
GVL: Graft-versus-leukemia
HSCT: Hematopoietic stem cell transplantation
T_eff_: Effector T cell
T_reg:_: Regulatory T cell
nT_reg_: Naturally occurring T_reg_
iT_reg_: Inducible T_reg_
DC: Dendritic cell
TEC: Thymic epithelial cell.
